# Replacement of Vena Cava up to the Right Atrium during Living Donor Liver Transplantation for *Echinococcus alveolaris*

**DOI:** 10.1155/2014/801657

**Published:** 2014-11-20

**Authors:** Fahri Yetişir, S. Murad Dogan, Ruslan Mamedov, Cuneyt Kayaalp, Sezayi Yilmaz

**Affiliations:** ^1^General Surgery Department, Atatürk Research and Training Hospital, Turkey; ^2^Liver Transplantation Institute, Inonu University, Malatya, Turkey; ^3^General Surgery Department, İzmir Research and Training Hospital, Turkey; ^4^Azerbaijan Medical University, Baku, Azerbaijan

## Abstract

Management of advanced stage of *Echinococcus alveolaris* is a very difficult procedure. Surgical treatment like resection and liver transplantation is accepted procedure nowadays. Here we presented a case report of *Echinococcus alveolaris* which invaded the inferior vena cava up to the right atrium and surrounding tissues. This patient underwent living donor liver transplantation with replacement of inferior vena cava up to the right atrium with cryopreserved cadaveric aortic graft. This procedure is very difficult but it is a life-saving chance for patients in advanced cases of *Echinococcus alveolaris*.

## 1. Introduction

Human alveolar* Echinococcus* is a potentially fatal, chronic, and progressive disease [1–3]. The treatment of EA is very difficult, because patients are coming to surgical centers with advanced stages of disease. In these cases partial liver resection can be performed in only 35% of patients [3]. Liver transplantation is only life-saving treatment of advanced stages of* Echinococcus alveolaris* [4]. Living donor liver transplantation (LDLT) has been also used to treat advanced stage EA, because of the rarity of cadaveric liver donor. In most cases EA may invade surrounding tissues including major vessel. Because of that, in these cases resection and replacement of major vessel are often unavoidable. That could force the surgeon to perform complicated surgical technique [5].

Herein we report a case of a patient with advanced stage EA invading the surrounding tissues and IVC up to the right atrium. This patient was treated with living donor liver transplantation with replacement of inferior vena cava up to the right atrium with cryopreserved cadaveric aortic graft.

## 2. Case Report

**Figure 1:**
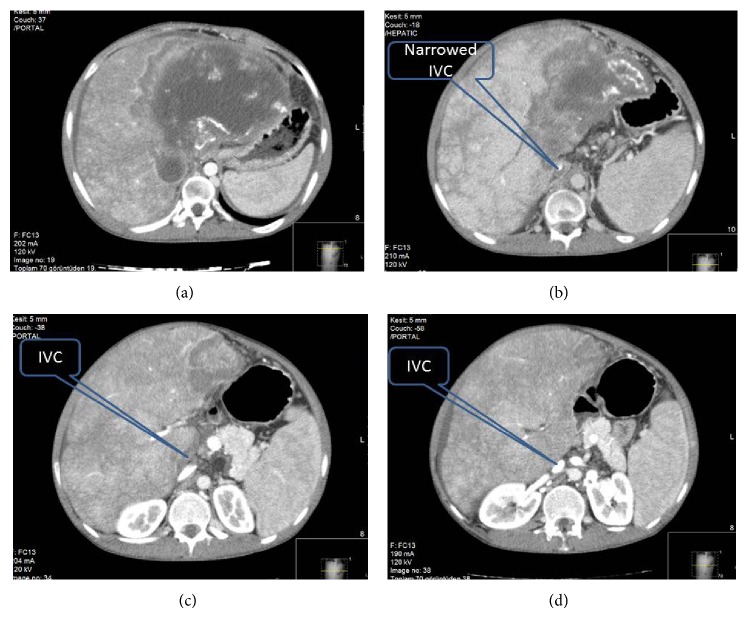
CT view of preoperative liver with (a) large mass filling almost all liver and IVC is nonvisible. (b) Invasion and compression of IVC by EA are seen. (c) Compression to surrounding tissues and organs by EA. (d) Compression and invasion of IVC extending up to renal vein level.

A 25-year-old young man was referred to our center with serious abdominal asit, lower extremities edemas. Indirect echinococcal haemagglutination test was positive and INR level was 1.7. Abdominal computerized tomography (CT) showed that the liver which was compressing the other abdominal content was occupying the entire abdomen and pelvis, in size of (38 × 29 cm). A large mass in the liver was compressing and invading the inferior vena cava and completely obstructing retrohepatic IVC (Figure 1).

Because of advanced stage EA (wide spread parasitic dissemination and liver deficiency), living donor liver transplantation was chosen as the method of definitive treatment and the patient's brother decided to be the donor.

**Figure 2:**
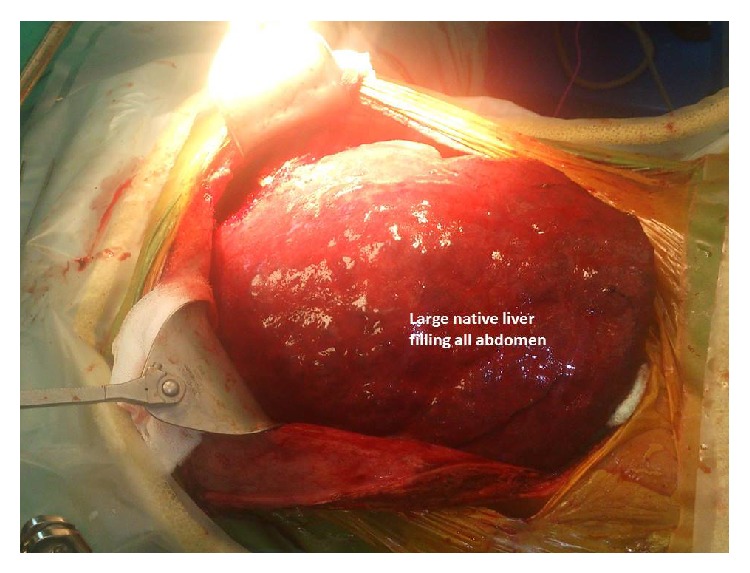
Large native liver filling the entire abdomen is seen.

**Figure 3:**
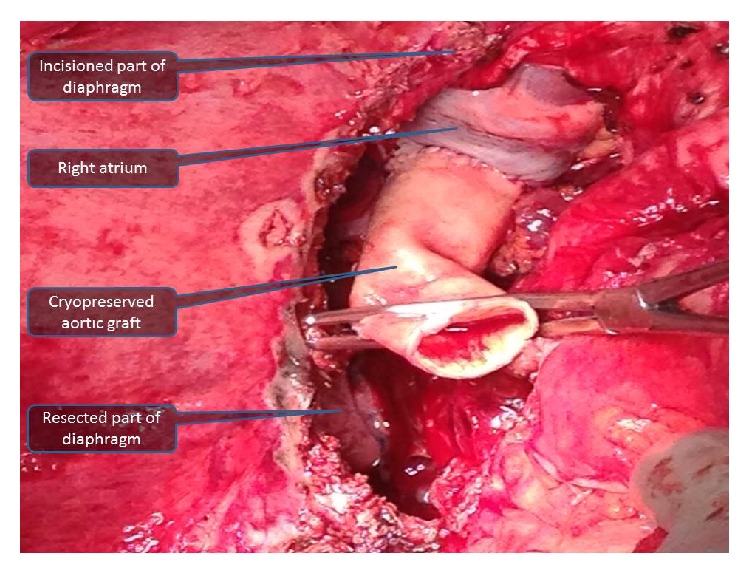
The view after diaphragm resection and the anastomosis between right atrium and first part of cadaveric aortic graft is seen.

Benz incision was used. Large liver with alveolar disease extended to pelvis and had strong adhesions with the surrounding tissues, anterior abdominal wall, right hemidiaphragm, colon and small intestine, and the retrohepatic part of inferior vena cava up to the right atrium that made recipient hepatectomy more difficult than other cirrhotic livers (Figure 2). In particular, strong invasion to the diaphragm and inferior vena cava extending in cephalic destination forces us to perform caval vein resection until right atrium and resection a left suprahepatic part of diaphragm (Figure 3). After IVC resection deposits of EA were seen on upper end of IVC, upper clamp was reclamped from one more centimeter upper and reexcision of invaded part of IVC was performed. 4-5 minutes after this procedure, a quick decline in arterial blood pressure occurred and hart bate on left diaphragmatic side had not been felt. After conforming hemopericardium, pericardiotomy was performed in seconds. The hematoma was evacuated and reclamped from atrium. After all of them hemodynamic status of patient improved.

**Figure 4:**
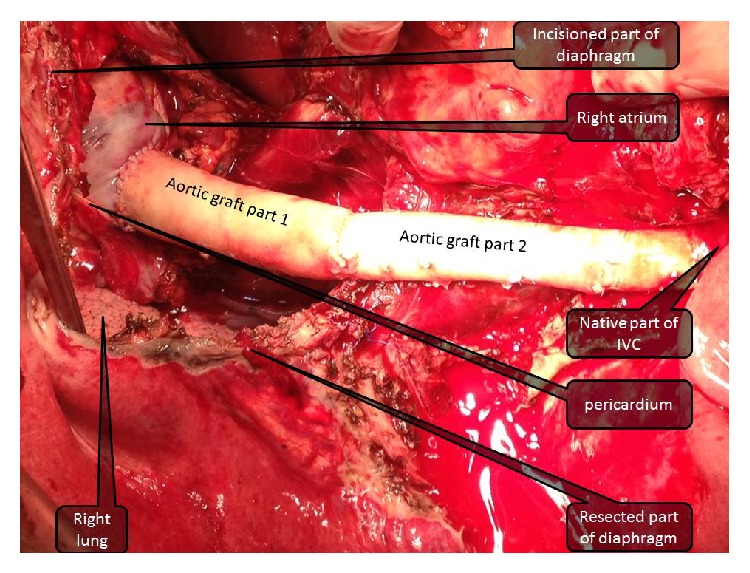
Incised pericardium and total replacement of retrohepatic part of vena cava (from renal vein to right atrium) are seen.

**Figure 5:**
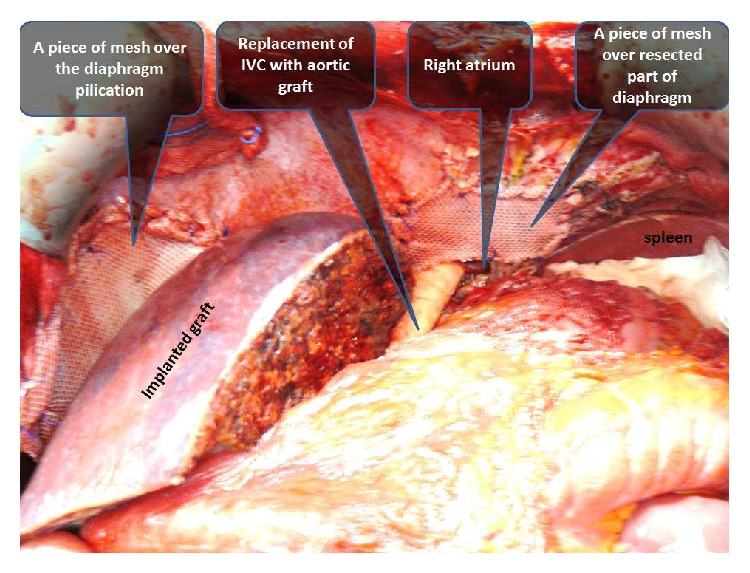
Final view after implantation. Diaphragm plication with mesh is seen.

**Figure 6:**
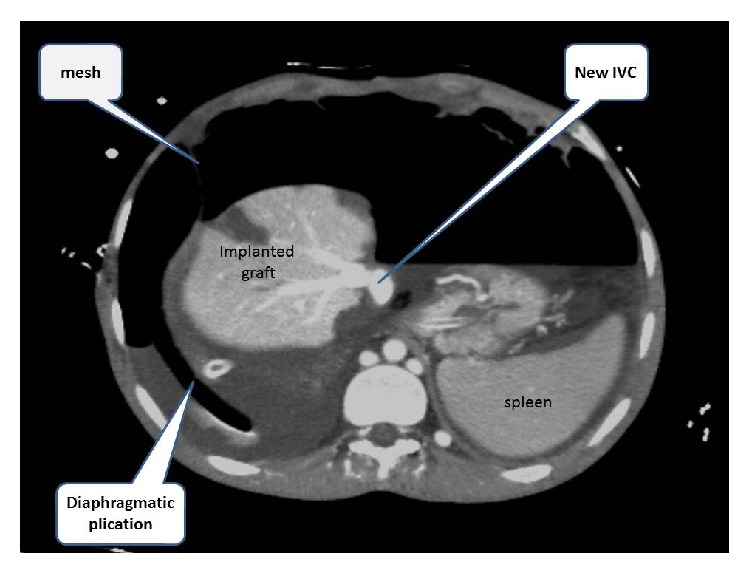
CT view after living donor liver transplantation. Diaphragmatic plication and new IVC are seen.

For performing this procedure, we needed to make a curvilinear incision through the tendinous part of the diaphragm near the excisioned part to get expose the pericardium. Replacement of inferior vena cava was done from the suprarenal part of IVC up to the right atrium by cryopreserved cadaveric aortic graft with end-to-end anastomosis (Figures 3 and 4). Defect in diaphragm was repaired with using mesh (Figures 5 and 6).

Donor's right lobe of liver with middle hepatic vein was transplanted and graft/recipient weight ratio was more than 1%. Venous and arterial reconstructions were carried out with a standard method. During back-table procedure, we used cryopreserved venous graft surrounding middle and right hepatic veins with aim to make all-in-one orifice. This provided us an easier and large anastomosis with new vena cava. End-to-end portoportal anastomosis was done. Arterial anastomosis was performed with ×6.5 surgical loupes by separated sutures. Duct-to-duct biliary anastomosis was performed, with an external tube replacing through the cystic duct. Because of the large space after removing of native liver, the newly created arteries and portal vein was under tension. For resolving this problem we used retrohepatic part of diaphragm plication with mesh (Figures 5 and 6). He was discharged on day 25 after operation.

## 3. Discussion

Despite the available multiple treatments for advanced stage EA including medical therapy [6], the surgical technique remains the only radical treatment for this pathology. Radical surgical treatment like liver transplantation is more preferable for treatment of EA, because palliative resection does not offer advantages when compared with conservative treatment [3]. The more that palliative surgery is often done because of the impossibility of complete removal of the parasite due to the spread it on the irresectable structures [5, 7].

There are several reports for liver transplantation in patients with EA, especially from endemic regions. Thus in 2009 Moray et al. reported two cases of liver transplantation for advanced EA and one of them had operation with resection and replacement of IVC with PTFE graft [4]. In our center previously, we performed resection and replacement of IVC during living donor liver transplantation to treat Budd-Chiari syndrome associated with hydatid cyst. Retrohepatic part of IVC in this patient was also replaced with cadaveric aortic graft [5, 8]. This report is the third case of complicated stage EA, which was treated by living donor liver transplantation. The difference in this case from our previous case was extending invasion of IVC until the right atrium. There are some reports that describe surgical technique for replacement of IVC until the right atrium. Thus, in 2012 Wise et al. presented a case of a patient with giant primary synovial sarcoma with invasion to the inferior vena cava extending to the right atrium with caval reconstruction under cardiopulmonary bypass and circulatory arrest. They performed bovine pericardial patch reconstruction of the IVC [9].

In 2013 Mancuso et al. reported a case of a patient with Budd-Chiari syndrome with replacement of IVC including intrapericardial part with a caval homograft [10].

We used this technique for management of advanced stage of EA during living donor liver transplantation and we did not need to perform this operation under cardiopulmonary bypass and circulatory arrest. Additionally for resolving tension of anastomoses we made diaphragm plication with mesh. According to our knowledge, these are new techniques for management of advanced stage EA.

Ex vivo liver resection and autotransplantation have been used successfully in suitable EA cases, but in our case, there was no disease-free, healthy liver tissue present to be used in autotransplantation.

Although some authors think that shaping of the hepatic veins of the graft and reconstruction with right atrium without reconstruction of retrohepatic vena cava would not be necessary when the collateral circulation was sufficient for compensation, we preferred to reconstruct retrohepatic IVC.

## 4. Conclusion

Living donor liver transplantation with caval replacement up to the right atrium is a very difficult operation but it is a life-saving chance for patients with EA invading IVC up to the atrium.
